# Prognostic Evaluation of Disorders of Consciousness by Using Resting-State fMRI: A Systematic Review

**DOI:** 10.3390/jcm13195704

**Published:** 2024-09-25

**Authors:** Maria Le Cause, Lilla Bonanno, Antonella Alagna, Carmen Bonanno, Jolanda De Caro, Anna Lisa Logiudice, Patrizia Pollicino, Francesco Corallo, Simona De Salvo, Carmela Rifici, Angelo Quartarone, Silvia Marino

**Affiliations:** IRCCS Centro Neurolesi Bonino Pulejo, 98124 Messina, Italy; maria.lecause@irccsme.it (M.L.C.); antonella.alagna@irccsme.it (A.A.); carmen.bonanno@irccsme.it (C.B.); jolanda.decaro@irccsme.it (J.D.C.); annalisa.logiudice@irccsme.it (A.L.L.); patrizia.pollicino@irccsme.it (P.P.); francesco.corallo@irccsme.it (F.C.); simona.desalvo@irccsme.it (S.D.S.); carmela.rifici@irccsme.it (C.R.); angelo.quartarone@irccsme.it (A.Q.); silvia.marino@irccsme.it (S.M.)

**Keywords:** consciousness, minimally conscious state, resting-state fMRI, vegetative state, cognitive rehabilitation, recovery of consciousness

## Abstract

**Background:** This review focuses on the prognostic role of resting-state functional magnetic resonance imaging (fMRI) in disorders of consciousness (DOCs). Several studies were conducted to determine the diagnostic accuracy in DOC patients to identify prognostic markers and to understand the neural correlates of consciousness. A correct diagnosis of consciousness in unresponsive or minimally responsive patients is important for prognostic and therapeutic management. Functional connectivity is considered as an important tool for the formulation of cerebral networks; it takes into account the primary sensorimotor, language, visual and central executive areas, where fMRI studies show damage in brain connectivity in the areas of frontoparietal networks in DOC patients. **Methods:** The integration of neuroimaging or neurophysiological methods could improve our knowledge of the neural correlates of clinical response after an acquired brain injury. The use of MRI is widely reported in the literature in different neurological diseases. In particular, fMRI is the most widely used brain-imaging technique to investigate the neural mechanisms underlying cognition and motor function. We carried out a detailed literature search following the relevant guidelines (PRISMA), where we collected data and results on patients with disorders of consciousness from the studies performed. **Results:** In this review, 12 studies were selected, which showed the importance of the prognostic role of fMRI for DOCs. **Conclusions:** Currently there are still few studies on this topic. Future studies using fMRI are to be considered an added value for the prognosis and management of DOCs.

## 1. Introduction

A disorder of consciousness (DOC) refers to a state of dissociation between wakefulness and consciousness, and can be found at different levels: coma, vegetative state or unresponsive wakefulness syndrome (VS/UWS), minimally conscious state (MCS) and emergence from a minimally conscious state (eMCS) [1,2,3]. A coma is the result of a severe brain injury, in which patients are no longer able to wake up (e.g., eyes closed even when stimulated) and are unaware of themselves and their environment. There are many uncertainties in the assessment and treatment of DOC patients. Patients in a coma have a complete absence of the arousal system, with no spontaneous eye opening and an inability to be aroused by the application of strong sensory stimulation. A VS is characterized by the complete absence of behavioral signs of environmental or self-awareness [4,5]. Diagnostic criteria for an MCS are distinguished from a VS by the presence of behaviors associated with conscious awareness [6]. Functional magnetic resonance imaging (fMRI) is a very powerful method for investigating brain responses to cognitive tasks. Resting-state fMRI (rs-fMRI) also has advantages in signal acquisition. The resting state (RS) paradigm is not difficult to perform experimentally, as the active contribution of the patient is not required [7]. Some studies have investigated prognostic criteria based on clinical characteristics: etiology, duration of injury, nutritional level and complications have been reported to be associated with clinical outcomes in DOC patients [8,9,10]. The increasing use of this method is due to its flexibility, availability, high spatial resolution, relatively high temporal resolution, and the absence of ionizing radiation or the need for external contrast agents. As shown in the literature, an fMRI assessment can objectively determine whether an unresponsive patient is conscious, even in the absence of explicit verbal or motor responses [11,12]. Moreover, functional connectivity is considered as an important tool for the formulation of cerebral networks; it takes into account the primary sensorimotor, language, visual and central executive areas. fMRI studies show damage in brain connectivity in the areas of frontoparietal networks in DOC patients [13,14,15]. A neuroimaging tool could open a space for analyzing residual neurofunctional activity in the absence of detectable behavioral responses in DOC patients [16]. The diagnosis of a DOC is based on clinical observations and standardized neurobehavioral assessment scales, such as the Coma Recovery Scale-Revised (CRS), Levels of Cognitive Functioning (LCFs), Glasgow Outcome Scale Extended (GOSE), and Glasgow Coma Scale (GCS), which refer to an individual’s ability to respond to both external and internal stimuli in an integrated manner. Specifically, the CRS-r is used to assess the level of consciousness of DOC patients [17,18]. The LCF involves behavioral observation to evaluate the structure and function at different levels of consciousness [19]. The GOSE has become one of the most widely used instruments to assess global disability and recovery after a traumatic brain injury [20]. The GCS provides a structured method for assessing the level of consciousness, with its derived sum score scoring system applied in research and adopted in intensive care units [21,22]. Neurobehavioral assessment scales require standardized scoring and the ability to detect subtle signs of consciousness [23,24]. The objective of this review was to evaluate the prognostic utility of rs-fMRI in patients with DOCs in order to evaluate the role of fMRI as an objective and quantitative method to assess prognostic markers in DOC patients. Given the complexities and uncertainties in assessing and treating DOCs, this review aimed to examine how rs-fMRI could enhance our understanding of the different levels of DOCs, including a coma, VS/UWS, MCS, and eMCS. This review explored the potential of rs-fMRI to provide objective measures of consciousness and brain connectivity, thereby improving diagnostic accuracy and prognostic predictions. Additionally, it will consider the integration of rs-fMRI with the existing neurobehavioral assessment to better define clinical evaluations and outcomes in DOC patients.

## 2. Materials and Methods

The process of literature exploration adhered to the guidelines set out by the Preferred Reporting Items for Systematic Reviews and Meta-Analyses (PRISMA) framework, which is designed to help report systematic reviews trasparently and use explicit, systematic methods to collect and summarize the results of studies that addressed a specific topic. In addition, our systematic review was registered in the international prospective register of systematic reviews called PROSPERO, which accepts qualitative and individual participant data reviews; this registration helps to avoid duplication and reduces the opportunity for reporting bias by enabling a comparision of the complete review with the planned protocol. This review was assigned a unique identifier (ID: CRD42024503515).

### 2.1. Information Sources and Search Approach

A comprehensive search of several databases, including PubMed, Web of Science and the Cochrane Library, was conducted to gather relevant information. This search included articles up to January 2023. The eligibility criterion for articles according to the PICO scheme was the 2013–2023 time frame, thus covering 10 years of research. In particular, the population considered included males and females, with additional filters for adults aged 19+. Articles in English dealing exclusively with the human species were included, while review studies were excluded. The following search terms that appeared in the title or abstract of the article were used: (“disorders of consciousness” [MeSH terms] or “resting state fMRI” [all fields] or “Coma Recovery Scale-Revised” [all fields]). Free full text articles were also searched. Initially, individual keyword searches yielded many results, which were further refined by combining the words “disorders of consciousness and resting state fMRI” or “Coma Recovery Scale-Revised and resting state fMRI”, which significantly narrowed the search. Keywords present in abstracts and free full-text articles were considered. Each database used different filters: Pubmed used the described filter, Web of Science used [open access, all fields, quick add keywords, publication years] and Cochrane Library used [title-abstract-keyword].

### 2.2. Selection and Data-Gathering Process

**Figure 1:**
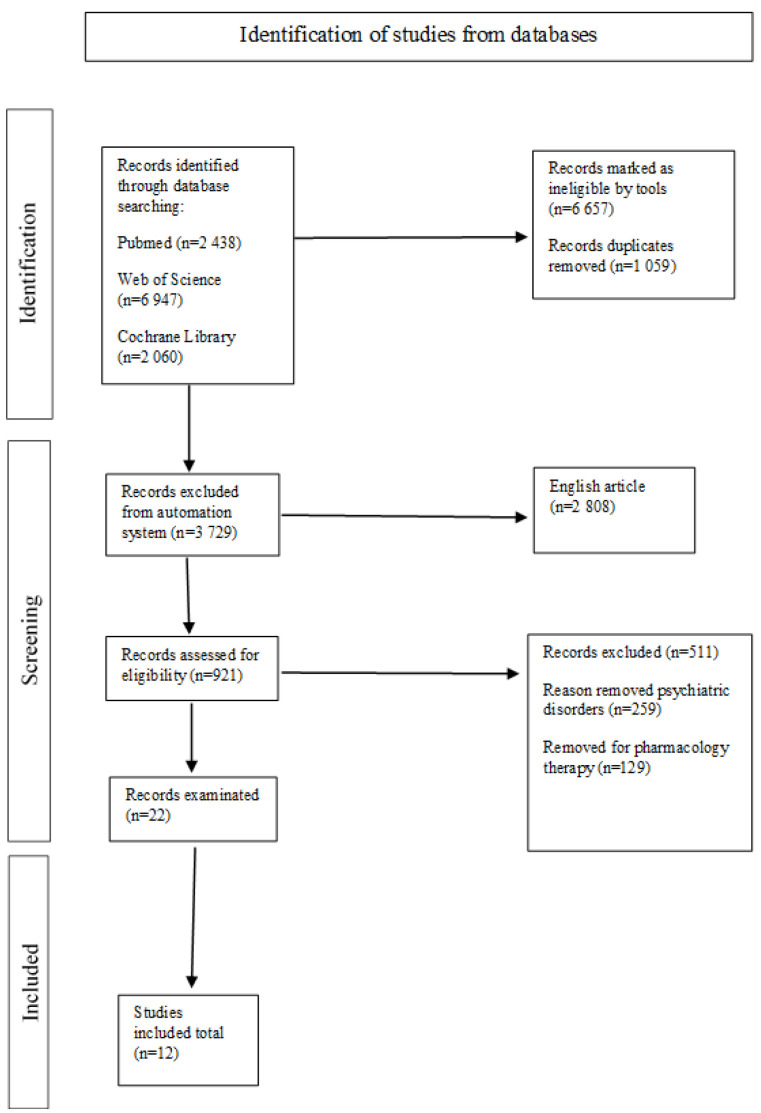
PRISMA Flowchart of identification, screening, eligibility and inclusion of selected articles. The flowchart shows the details of our search in a diagram. We selected 11,445 articles for comprehensive review in several electronic databases; 12 studies met the inclusion criteria.

The entire process of study selection, including the number of studies screened; assessment for eligibility; and finally, inclusion in the review, along with an explanation for exclusion at each stage, is presented in Figure 1, which illustrates the PRISMA flowchart of the study selection process. Data from the included studies were extracted using a custom spreadsheet with various characteristics. Duplicate articles found on the three platforms were eliminated. Articles were selected using an automated screening process and the work of researchers.

### 2.3. Data Elements

Studies were included in this review if they met the following criteria: relevant population details (studies in adult patients, exclusion of psychiatric disorders), use of CRS and rs-fMRI, and relevant brain regions (mesio-frontal, medial region, meso-frontoparietal cortex). The search and assessment of studies were carried out in several stages (each study was triple checked for inclusion) by the same researcher who performed the keyword search. This preliminary work took about one month. ROBIS, which was the first rigorously designed tool to assess the risk of bias in systematic reviews, was used, and in this review, the risk of bias was low.

## 3. Results

Selected research findings in this review reported diagnoses based on observational clinical tests and fMRI experiments. The outcome results are different; in fact, patients in a VS are less likely to show significant improvement. fMRI and CRS-R correlate active brain areas with improvement and damaged areas. In particular, research showed that the recovery of some complex behavioral responses exists [25]. The significant relationship between patients’ CRS-R scores and the level of deactivation in the medial parietal and medial frontal regions confirms the hypothesis that the capacity for deactivation is associated with the level of consciousness [26]. Brain activity could help to discern whether wakefulness in a VS is also accompanied by partial awareness, as occurs in an MCS, which has important prognostic implications [7]. The use of rs-fMRI showed the activation of areas, especially in MCS patients, in contrast with patients in a UWS [26]. There is a consensus that different brain regions could be impaired in DOCs [27,28,29]. Frequently, the visual [30] and auditory [7] areas are implicated in DOCs. Additionally, brain activity could be used to predict clinical scores in DOC patients [29]. Conventional fMRI task-related studies showed no significant differences in functional activation between VS and MCS groups, which highlights the potential for functional connectivity MRI (fcMRI) as a clinical tool for differential diagnosis in DOCs [27]. Resting-state neuroimaging assessments reveal specific brain organization that primarily involves the posterior cingulate and adjacent pre-cuneus cortex, as well as the anterior cingulate cortex and meso-frontal regions (notably the default mode network) [31]. These findings have significant clinical implications for predicting outcomes. This is crucial for prognosis because despite the single assessment of consciousness in these patients, the initial phase often involves rapid fluctuations in the level of consciousness. This emphasizes the importance of early and rigorous differentiation between a VS/UWS and an MCS for accurate prognostic information and therapeutic approaches [32]. To date, the limitations of fMRI for DOCs do not appear to be present because the research is still in the process of being developed; the sample of subjects was too small to obtain an important indicative value.

This section represents a a concise and precise description of the experimental results, their interpretation and the conclusions that can be drawn (Table 1 and Table 2).

## 4. Discussion

To date, there are still few studies on this topic, though the obtained results are promising. The clinical tools currently used in practice permit an accurate diagnosis, but fMRI in particular has prognostic value in the early diagnosis of consciousness impairment, which may provide important information to guide medical decisions. The combination of fMRI and CRS-R evaluation is the most very useful for perform appropriate interventions. For patients with DOCs who have associated motor, language and cognitive impairments, functional neuroimaging could accurately reveal conscious awareness. All conscious patients showed variable cognitive damage, and some also presented behavioral and psychological symptoms. Although some patients had residual motor and cognitive disabilities with late recovery, they exhibited varying degrees of functional recovery, which contributed to a better understanding of the disease’s course. However, research is still needed for a correct and standardized prognosis: the main limitation today is the large disparity and small sample size of patients [35]. These findings require further investigation with larger patient sample sizes. In these studies, all patients had a common etiology; in four studies, the VS patients showed significant clinical improvement by evolving to an MCS, while other VS and MCS patients remained clinically stable. The importance of brain activity and neuronal correlation in terms of prognostic information was evident in the remaining eight studies Table 2. In addition, the fMRI tool was of considerable importance since it provided data on preserved mental processes after a severe brain injury. In patients with DOCs, functional neuroimaging provides more detail on which areas of consciousness are active [35]. The CRS-R, as described above, is the only method that directly obtains the existing diagnostic criteria for a VS, MCS and MCS emergency in the administration and scoring scale; however, in the assessment of persons with DOCs, command execution and functional communication may change over time and even during the same day. The recovery of consciousness can occur over an undefined period of time in disorders of consciousness, where it ranges from acute to subacute and chronic stages. The use of neuroimaging to better characterize brain processes in the acute and chronic phases could allow for personalized therapies. The development of these treatments and the evaluation of their efficacy can reveal evidence of therapeutic responses before behavioral effects are observed. Quantifiable general and regional changes in brain tissue, structural integrity, metabolism and functional connectivity were observed in patients with DoCs, as shown by diagnostic and prognostic biomarkers. In summary, rs-fMRI offers significant potential for improving the diagnostic accuracy and prognostic predictions in DOC patients. The integration of rs-fMRI with neurobehavioral assessment scales, such as the CRS-R, could improve clinical evaluations and outcomes, thus providing a more comprehensive understanding of consciousness levels and guiding therapeutic interventions. Further research with larger, more standardized patient samples is necessary to confirm these findings and to enhance the prognostic utility of rs-fMRI in clinical practice. Based on this evidence, the guidelines recommended “wherever feasible, consider PET, resting state, fMRI, EEG paradigms to complement behavioral assessment for detecting patients with covert cognition or with high probability to recover”.

## 5. Conclusions

This review provides an overview of the prognostic role of resting-state fMRI in disorders of consciousness. Several advances have been made in the diagnosis and prognosis of DOCs, but there is still much uncertainty in this area. As there is evidence that some patients with DOCs have the potential for the recovery of consciousness and neurological function, all possible diagnostic, prognostic and therapeutic approaches should be considered to support the recovery of consciousness in patients with DOCs [36]. Prognostic models may be useful for clinicians in making individual treatment decisions [37]. The 12 studies reviewed exemplify the importance of connectivity, which enables the accurate assessment of patients with disorders of consciousness. As expressed above, the results still do not allow for a solid line regarding prognosis, but this highlights how studies need to be continued and how the right methodology is being used today.

## Figures and Tables

**Table 1:** Summary of studies on disorders of consciousness.

Authors	Sample	Objective	Country	Diagnosis	Etiology	Time Since Diagnosis	Prognostic Factors	Data Acquisition	Treatment	Results
Estraneo, A. et al., 2014 [25]	13 patients	Assessment of patients’ clinical condition and neuropsychological functioning.	Italy	7 MCS 6 eMCS	6 anoxia 6 closed head injury 1 hemorrhagic stroke	More than 12 months after onset in patients with head injury and more than 6 months post-onset in patients with non-TBI	Age, N20 cortical component preserved on one side	CRS-R Neuroimaging	NA	Assessment of the preserved N20 cortical component of somatosensory potentials evoked on at least one side revealed that 5 were still minimally conscious; 6 recovered some complex behavioral responses to the environment.
Crone, J.S. et al., 2011 [26]	25 patients	To demonstrate DOCs that occurred in survivors of severe brain injury. Deactivation of the DMN is thought to reflect disruption of introspective processes.	Austria	8 MCS 17 UWS	12 traumatic 13 hypoxic	About 2 years	Correlation between the deactivation in the DMN and the level of consciousness.	CRS-R fMRI	Not applicable.	17 patients (6 MCS patients; 11 UWS patients) showed no significant deactivation pattern. Deactivation in the medial regions of DMN was reduced in an MCS and absent in a UWS.
Kotchoubey, B. et al., 2013 [27]	12 patients	In diagnosing disorders of consciousness, it is important to differentiate vegetative states from minimally conscious states.	Germany	6 MCS 6 VS	1 traumatic 7 anoxic 4 hemorrhagic	About 3 years	NA	CRS-R Functional connectivity	NA	Lack of connectivity between the posterior cingulate cortex and medial prefrontal cortex indicated that the patients had a loss of consciousness.
Stein, S. et al., 2015 [28]	27 patients	The observation of a disruption in the long-range communication between neurons is a major deficit in DOCs when observing network impairment and correlation with clinical deficits.	France	12 VS 11 MCS 4 other	13 traumatic 15 anoxic	About 2 years	Specific pattern of connectivity from the pre-cuneus and posterior cingulate cortex	CRS-R fMRI	NA	Lack of connectivity between the posterior cingulate cortex and medial prefrontal cortex indicated that the patients had a loss of consciousness.
Cao, B. et al., 2021 [29]	17 patients	Deficit structure predicts clinical outcomes in patients with disorders of consciousness using rs-fMRI in the resting state	China	11 VS 6 MCS	5 TBI 12 HIE	NA	Intrinsic brain activity imaging indicator	CRS-R fMRI	NA	DOC patients showed a time delay. Abnormalities in the precentral gyrus, pre-cuneus, middle cingulate cortex and postcentral gyrus.
Di Perri, C. et al., 2016 [31]	58 patients	Study on DOCs to identify neural network dysfunction using imaging.	Belgium	21 VS 24 MCS 13 eMCS	29 TBI 14 anoxic 7 TBI/anoxic 8 other	About 5 years	NA	CRS-R Neuroimaging	NA	Resting-state neuroimaging suggests a specific brain organization that primarily involved the posterior cingulate cortex and adjacent pre-cuneus, as well as the anterior cingulate cortex and meso-frontal regions, which is known as the default mode network.
Medina, J.P. et al., 2022 [30]	109 patients	Clarification of relevance to clinical evaluation	Italy	34 MCS 65 VS 10 SD	33 traumatic 39 vascular 37 anoxic	About 2 years	NA	CRS-R Neuroimaging techniques	NA	Correlative findings only suggest that visual networks could provide additional information about visual function in DOCs and may be functionally relevant to the patient’s level of consciousness.
Wang, Y. et al., 2022 [33]	39 patients	Functional changes in regional brain connectivity and activity were examined in this study.	China	14 MCS 25 VS	14 TBI 15 ICH 10 others	NA	NA	CRS-R Resting-state fMRI	NA	The VS/UWS group showed reduced functional connectivity between the DMN and the SN, between the SN and the ECN, and within the SN compared with the MCS group.
Faugeras, F. et al., 2018 [32]	67 patients	Determine whether patients diagnosed early on as minimally conscious (MCS) have a better prognosis.	France	34 MCS 33 VS	12 TBI 21 anoxia 14 ICH 20 other	About 5 years	Early diagnosis	CRS-R	NA	Early accurate clinical diagnosis of a VS/UWS or MCS had a strong prognostic value for survival and recovery of consciousness.
Yu, Y. 2020 [34]	51 patients	To develop a prognostic model of DOC recovery using a combination of laboratory parameters and resting functional magnetic resonance imaging (fMRI).	China	34 MCS 17 VS	TBI detail not specified	About 1 year	Information from neuroimaging and clinical parameters	CRS-R fMRI	NA	Combining information from the meso-frontoparietal cortex and the lower part of the brain may be a strategy to provide a more accurate prognosis.
Marino, S. et al., 2017 [7]	50 patients	Differences in brain activation in a large sample of vegetative state (VS) and minimally conscious (MCS) patients using functional magnetic resonance imaging (fMRI).	Italy	27 MCS 23 VS	25 TBI 11 CVA 14 other	4 to 7 months	fMRI could be a potentially reliable marker	CRS-R fMRI	NA	Significant bilateral brain activation in the primary auditory cortex during acoustic stimuli in patients with both VS and MCS.
Bodien, Y.G. et al., 2016 [35]	54 patients	Accurate diagnosis of the patient’s level of consciousness is critical to determining the treatment objectives, access to rehabilitation services and the prognosis markers.	USA	31 MCS 23 VS	TBI detail not specified	NA	NA	CRS-R fMRI	NA	The use of motor imagery to detect consciousness was not accurate.

**Notes:** CRS-R—Coma Recovery Scale-Revised, CVA—cerebral vascular accident, DMN—default mode network, DOC—disorder of consciousness, fMRI—functional magnetic resonance imaging, HIE—hypoxic ischemic encephalopathy, ICH—intracranial hemorrhage, MCS—minimally conscious state, SD—severe disability, TBI—traumatic brain injury, UWS—unresponsive wakefulness syndrome, VS—vegetative state, VS/UWS—vegetative state/unresponsive wakefulness state, NA—not applicable.

**Table 2:** Summary of importance differences regarding studies.

Authors	Improvement	Differences in Activation Status
Estraneo, A., et al., 2014 [25]	Recovery of some behavioural responses to the environment.	
Stein, S.; et al., 2015 [28]	VS/MCS group showed improvements.	
Marino, S., et al., 2017 [7]	Ten patients with VS had a significant clinical improvement, evolving into MCS.	
Bodien, Y.G., et al., 2016 [35]	Presence of improvement from VS/MCS.	
Crone, J.S., et al., 2011 [26]		Deactivation of the network in default mode as an indicator of compromised Consciousness.
Kotchoubey, B., et al., 2013 [27]		Activation status in different areas in VS/MCS.
Cao, B., et al., 2021 [29]		Brain activity detection.
Di Perri, C., et al., 2016 [31]		Neuronal correlation.
Medina, J.P., et al., 2022 [30]		Exploring the residual functional activity of patients’ brains in relation to clinical data.
Wang, Y., et al., 2022 [33]		Understanding the underlying neural mechanism of functional connectivity.
Faugeras, F., et al., 2018 [32]		Importance of an early and strict distinction between VS/MCS.
Yu, Y., 2020 [34]		Activity visualisation models.

## Data Availability

Not applicable.

## Abbreviations

The following abbreviations are used in this manuscript:

MDPI	Multidisciplinary Digital Publishing Institute
DOAJ	Directory of open access journals
TLA	Three-letter acronym
LD	Linear dichroism
